# Measurement data from sample plots characterizing three development stages of a subalpine spruce forest and describing the bird assemblages associated with them

**DOI:** 10.1016/j.dib.2020.105473

**Published:** 2020-04-05

**Authors:** Małgorzata Bujoczek, Leszek Bujoczek, Judyta Rybicka

**Affiliations:** a University of Agriculture in Krakow, Faculty of Forestry, Department of Forest Biodiversity, Al. 29 Listopada 46, Krakow 31-425, Poland; b University of Agriculture in Krakow, Faculty of Forestry, Department of Forest Resources Management, Al. 29 Listopada 46, Krakow 31-425, Poland

**Keywords:** Density, Volume, Stumps, Snags, Downed deadwood, Bird diversity, Ecological niches

## Abstract

The paper presents data on tree stands located in a subalpine spruce forest in the Gorce National Park (Western Carpathians, Poland) as well as on the birds inhabiting them in the breeding and nonbreeding seasons. The authors conducted stand measurements on a total of twenty-four sample plots, with eight plots established in each of the three studied forest development stages (mature, break-up, and growing-up) representing successive phases of subalpine forest development. The study area designated for each stage was 30 ha. The development properties of each study area resulted from both biotic and abiotic factors. Tree stands were characterized in terms of the tree layer, saplings, and deadwood. The types of deadwood measured were: standing entire dead trees, snags, stumps, and downed deadwood. In the case of birds, the presented data include species composition and pair density in the breeding season and individual bird density in the nonbreeding season. The authors used these data in a paper [1] examining the relationship between forest structural indicators and bird diversity in the three forest development stages. Other researchers can also utilize these data for the purpose of comparison with other areas representing similar forest communities or for studies on stand dynamics, especially in the face of climate change which may substantially affect those phenomena. Furthermore, the presented data may be useful in studies concerning the abundance and dynamics of bird assemblages and other organisms associated with the subalpine zone as well as in research devoted to the diversity of ecological niches. Such investigations may lay foundations for developing conservation guidelines for the ecosystems in question.

## Highlights

• Data on the characteristics of tree stands as well as the birds inhabiting them were collected from a subalpine spruce forest.
• The study area exhibited development properties resulting from both biotic and abiotic factors.
• Tree stands were characterized in terms of the tree layer, saplings, and deadwood.
• The data included bird species composition and pair density in the breeding season or individual bird density in the nonbreeding season.

**Specification table**

**Table:** 

Subject	Ecology
Specific subject area	Forest ecosystem characteristics, ecological niches, the presence of species with specific nesting and foraging requirements.
Type of data	Table Figures
How data were acquired	Field measurements of tree stands, bird counts conducted by the combined territory mapping method (the tools included: a tree caliper, GPS system, Vertex IV, Vortex Viper HD binoculars 10 × 42)
Data format	Raw
Parameters for data collection	The data concern three stages of subalpine forest development resulting from biotic and abiotic disturbances. The area designated for each stage was 30 ha. The authors subjectively delineated the boundaries of the study areas in such a way that the tree layer in each area was structurally homogeneous.
Description of data collection	The authors established eight sample plots in each stage. The plots were located at the nodes of a 200 *m* × 200 m grid. Each sample plot consisted of two concentric circles. Living trees and deadwood were measured on the larger one (0.05 ha), while the smaller one (0.01 ha) was used for counting saplings. In addition, birds were counted in each forest development stage in the years 2016–2017. Five counts took place in the breeding season (March–July) and three in the non-breeding season (October–January).
Data source location	Małgorzata Bujoczek, University of Agriculture in Kraków, Faculty of Forestry; Kraków, Poland
Data accessibility	Repository name: Mendeley data Direct URL to data: https://data.mendeley.com/datasets/kt8fz8mrt9/2
Related research article	Małgorzata Bujoczek, Judyta Rybicka, Leszek Bujoczek Effects of disturbances in a subalpine forest on its structural indicators and bird diversity. Ecological Indicators 112 (2020) 106126. https://doi.org/10.1016/j.ecolind.2020.106126

## Value of the Data

• The presented data concern ecosystems that are relatively scarce and require conservation. The authors collected stand data using statistical methods of forest inventory taking, which are widely applied in studies of forest ecosystems. We performed bird counts by means of combined territory mapping. The data can be further processed or compared with similar data for other ecosystems around the world.
• The data can be used by researchers dealing with the dynamics of forest communities, ornithologists, and ecologists, as well as by organizations and institutions responsible for forest management policies and conservation.
• The data can be expanded to other groups of organisms requiring specific ecological niches depending on the dynamics of forest ecosystems. Ornithologists can use them to determine behavioral adaptations to environmental change. Finally, they may be applied as a foundation for developing conservation guidelines for such ecosystems, which are particularly vulnerable to disturbances related to climate change.
• The data describe three successive development stages of a subalpine forest in conjunction with information about the species composition and abundance of the birds inhabiting them. They provide a comprehensive picture of the studied areas including parameters of the tree layer and regeneration, as well as qualitative and quantitative characterization of ecological niches associated with deadwood (size, tree species, decay stage, and type of deadwood).

## 1 Data

**Fig. 1:**
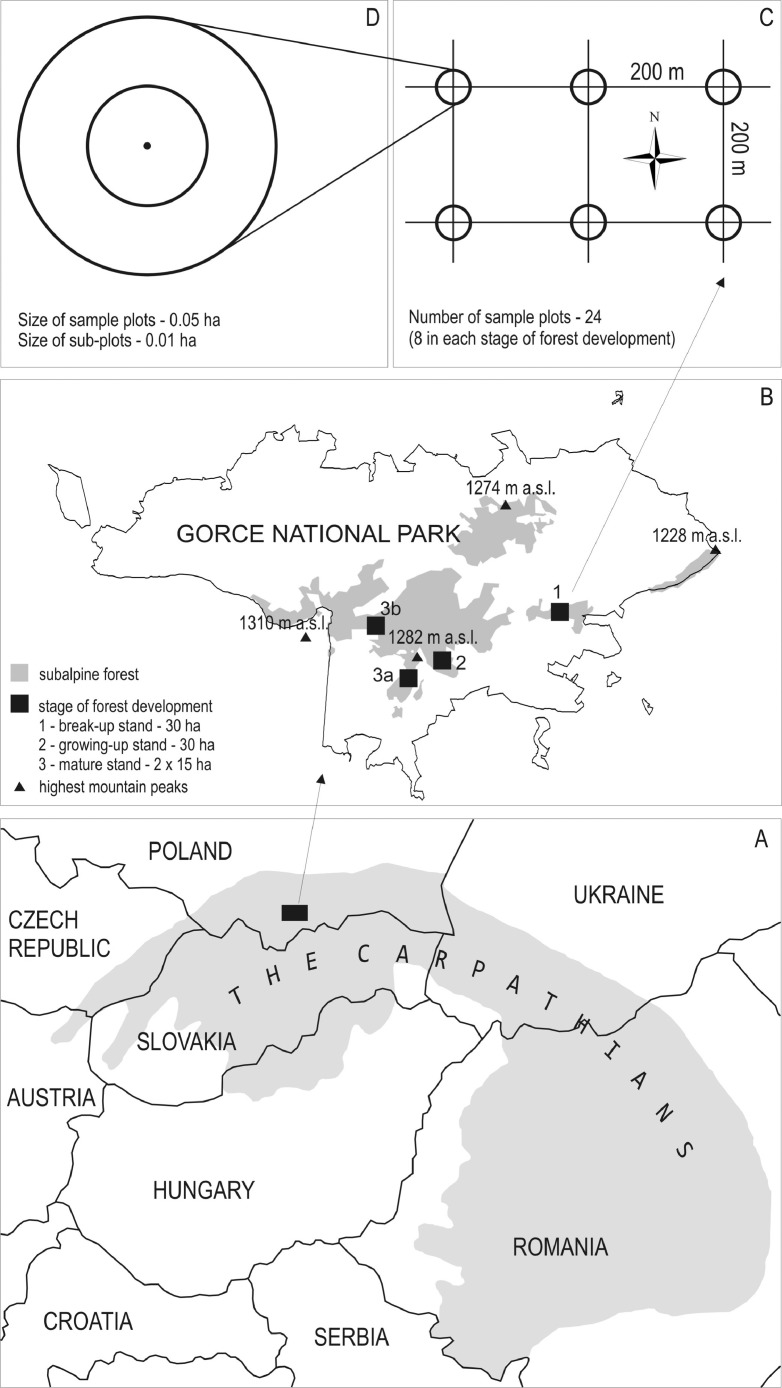
A. Location of the study area in the Carpathians, B. Location of the selected stages of forest development, C. Grid with sample plots, and D. Diagram of an individual sample plot.

**Fig. 2:**
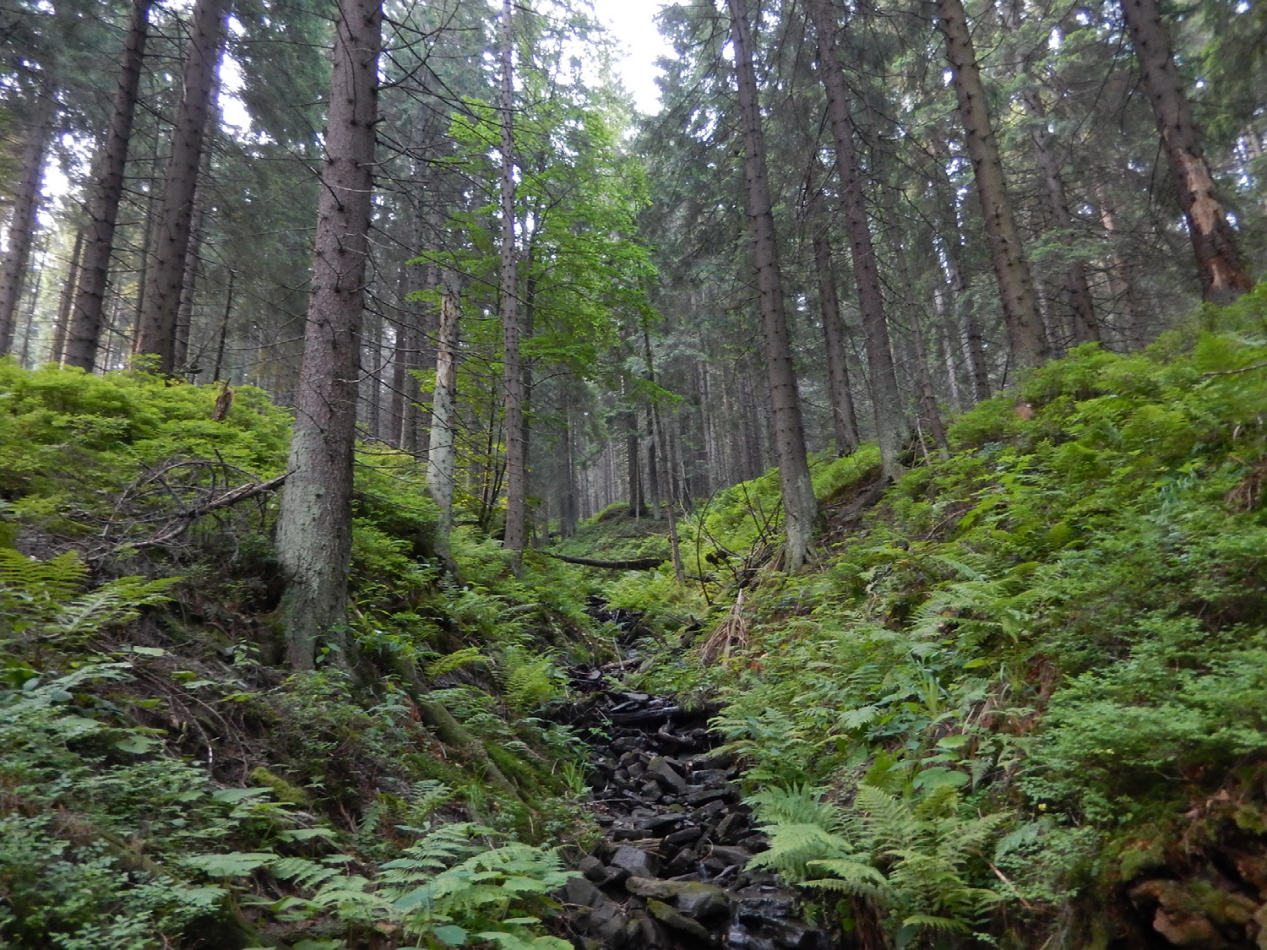
Mature stage of forest development.

**Fig. 3:**
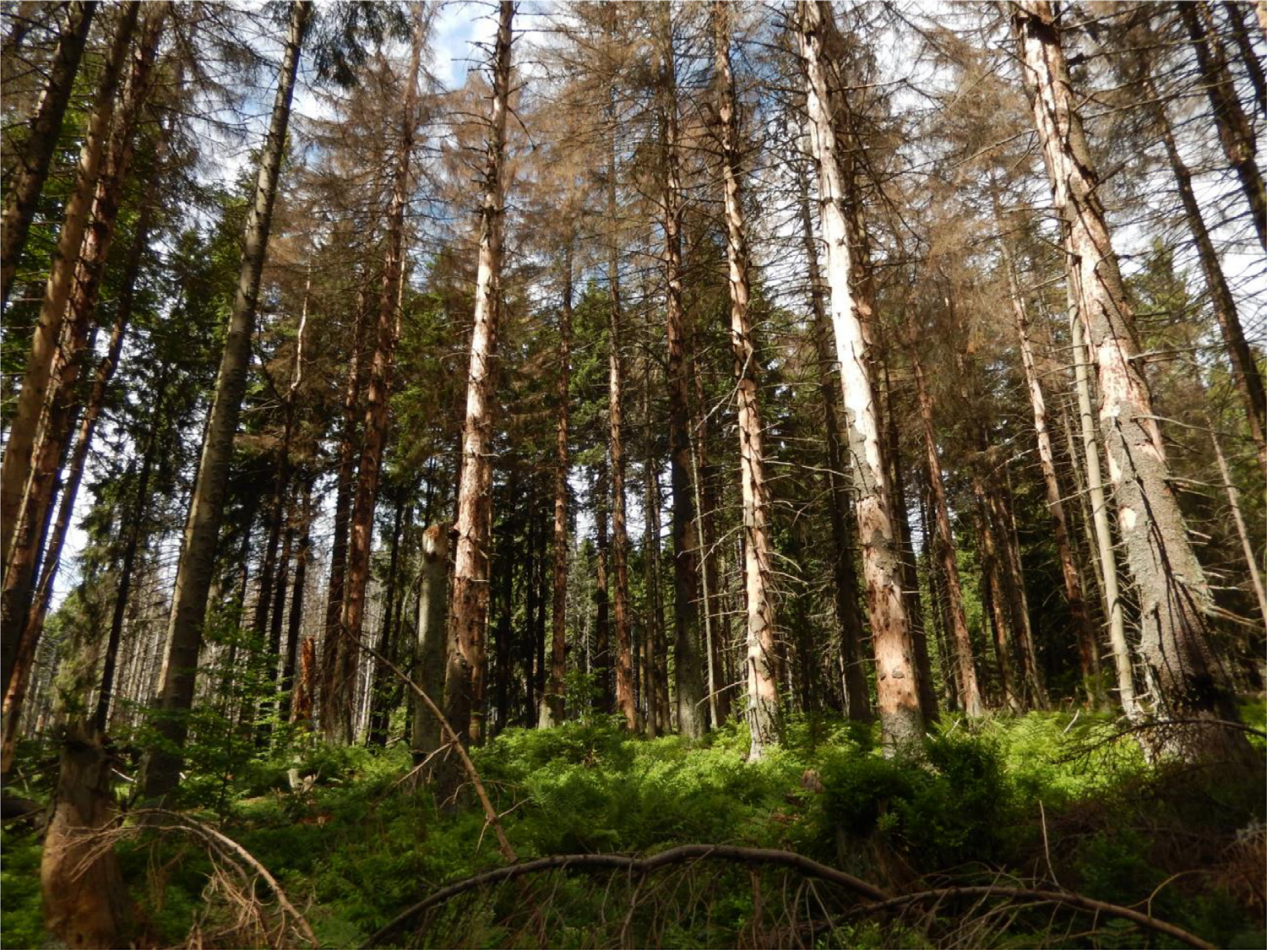
Break-up stage of forest development.

**Fig. 4:**
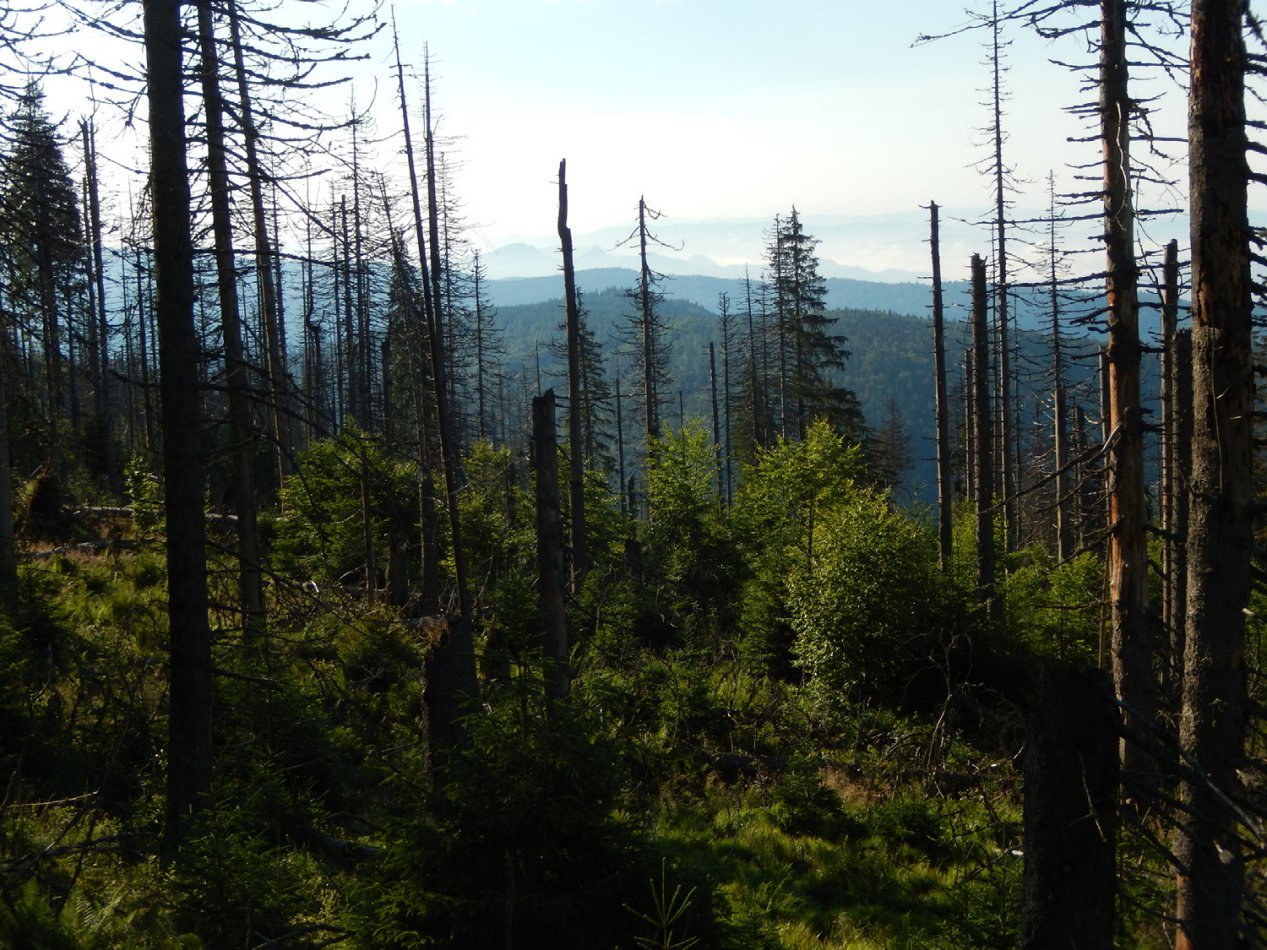
Growing-up stage of forest development.

The dataset contains measurement data on the tree layer, deadwood, and saplings collected from subalpine stands in three different development stages. An .xlsx file with those data was deposited at https://data.mendeley.com/datasets/kt8fz8mrt9/2. In addition, the manuscript contains five figures and one table. Fig. 1 depicts the location of the study area in the Carpathians, the location of the selected forest development stages, the distribution of sample plots, and a diagram of an individual sample plot. Fig. 2, Fig. 3, Fig. 4 present photographs of the three subalpine forest development stages. Fig. 4 contains a diagram schematically showing measurements of living trees and deadwood on sample plots. Table 1 lists the species composition and bird density in the nonbreeding season as well as the density of bird pairs in the breeding season in the three stages of subalpine forest development.

**Table 1:** Species composition and density of birds in the nonbreeding season/density of bird pairs in the breeding season in the three stages of subalpine forest development.

Species	Nonbreeding season			Breeding season		
	Growing-up	Mature	Break-up	Growing-up	Mature	Break-up
	in./10 ha	in./10 ha	in./10 ha	pair/10 ha	pair/10 ha	pair/10 ha
*Accipiter gentilis*	0.1	0	0	0	0	0
*Accipiter nisus*	0.3	0	0	0	0	0.3
*Anthus trivialis*	0	0	0	0	0	0.7
*Certhia familiaris*	0.1	0.4	0.2	0.7	1.3	1.3
*Columba palumbus*	0	0	0	0.3	0	0.3
*Dendrocopos major*	1.3	0.8	0.6	0.7	0	0.3
*Dryocopus martius*	0.1	0	0.2	0.3	0	0.3
*Erithacus rubecula*	0	0	0	0.3	1.7	1.7
*Fringilla coelebs*	0	0	0	0.7	2.0	1.7
*Garrulus glandarius*	0.1	0.3	0	0	0	0.3
*Glaucidium passerinum*	0.3	0.1	0	0	0	0.3
*Lophophanes cristatus*	0	0.4	0	0	1.7	1.0
*Loxia curvirostra*	0.1	0	0	1.0	1.3	0.7
*Nucifraga caryocatactes*	0	0	0	0.3	0	0
*Parus major*	1.8	1.6	1.0	0	0	0.3
*Periparus ater*	1.9	3.1	2.0	2.9	8.5	3.5
*Phoenicurus ochruros*	0	0	0	1.0	0	0.7
*Phoenicurus phoenicurus*	0	0	0	0.3	0	0.3
*Phylloscopus collybita*	0	0	0	1.3	1.3	1.0
*Phylloscopus trochilus*	0	0	0	0.3	0.3	0.3
*Picoides tridactylus*	0	0	0.9	0	0.3	0.7
*Poecile montanus*	0.8	1.4	0.4	1.0	0.3	1.0
*Poecile palustris*	0	0	0	0	0.3	0
*Prunella modularis*	0	0	0	0.3	0	0.7
*Pyrrhula pyrrhula*	0.6	0.8	0.7	1.0	1.0	0.7
*Regulus regulus*	0.1	4.2	1.1	0	1.7	0.3
*Sitta europaea*	0.3	0.2	0	0	0.7	0.3
*Spinus spinus*	0	0	0	0	0.3	0.3
*Sylvia atricapilla*	0	0	0	3.3	2.0	2.0
*Tetrao urogallus*	0.3	0	0	0.7	0	0.3
*Troglodytes troglodytes*	0.3	0	0	2.7	3.0	4.0
*Turdus merula*	0	0	0	0.3	0	0
*Turdus viscivorus*	0.1	0.1	0	0.3	0	0

The deposited .xlsx file with the measured data concerning three subalpine forest development stages contains three sheets. The first one, entitled “Stand,” presents measurements of living trees and deadwood carried out on twenty-four sample plots with an area of 0.05 ha each (column name – Number of sample plot). The column “Stage” specifies the stage of forest development for the sample plots: break-up (sample plots nos. 1–8), growing-up (9–16), or mature (17–24). The “Tree species” column provides the name of the identified tree species (or states “unspecified” for deadwood in advanced decay stages). The “Type” column describes the type of the measured element: living trees (Living) or deadwood (Entire dead trees, Snags, Stumps, Downed deadwood). The column “Diameter at breast height (DBH)” provides the DBH of living trees and snags. The columns “EndD1” and “EndD2” give diameters at the ends of downed deadwood, or the bottom and top diameters of snags and stumps. If downed deadwood did not have a circular cross-section, but was flattened as a result of decomposition, “EndH1” and “EndH2” values specify the height of such deadwood. The column “Height or length” contains the height of living trees, snags, and stumps or the length of downed deadwood within the boundaries of sample plots. The column “Decay class” gives the degree of deadwood decomposition on a five-point scale. The sheet “Canopy” contains the columns “Number of sample plot,” “Stage,” and additionally “Canopy closure,” which provides percentage canopy closure evaluated for the 0.5 ha plots. The third sheet, “Saplings,” supplies data from twenty-four sample plots with an area of 0.01 ha each, concerning the species composition and dimensions of saplings. This sheet contains the following columns “Number of sample plot,” “Stage,” “Tree species,” and three additional columns for sapling sizes (with the number of saplings per size class). If no saplings were found, zeros were entered, with the “Tree species” column left blank.

The species composition of the avifauna associated with the three studied subalpine forest development stages are given in Table 1. The mean numbers of birds observed during three counts on an area of 30 ha is provided for each species and each forest development stage in the nonbreeding season, while the number of bird pairs belonging to the various species is provided for the same areas in the breeding season.

## 2 Experimental design, materials, and methods

The authors collected the data in a subalpine spruce forest located in the Gorce National Park. The park with an area of 7029 ha was established in 1981 in the Western Carpathians, Poland (49°36′38″ N 20°03′45″ E) (Fig. 1). The subalpine forest together with the associated flora transition zone located between the montane and subalpine zone accounts for 27% of the park area [2]. Its vegetation, communities, and climatic conditions were described at length in Bujoczek et al. [1]. Since the beginning of the 1980s, subalpine forests have seen intensive changes caused by both biotic factors (mostly *Cephalcia alpina* and *Ips typographus*) and abiotic disturbances (windthrow, insufficient precipitation, declining water table, and high temperatures during the growing season). Those phenomena are likely to be associated with the global climate change. For the past several decades, mature spruce stands have been affected by intensive break-up processes [3]. Initially, in some subalpine forests the break-up stands were subjected to human intervention, but those areas have since come under strict protection. The subalpine forest of the Gorce National Park constitutes a mosaic of stands in different forest development stages. Forest patches of different sizes, in different development stages and with varying regeneration abundance, are interspersed with one another. Based on Korpel's classification [4,5] we distinguished the following three subalpine forest development stages: mature, break-up, and growing-up (Figs. 2–4). We designated a 30 ha study area for each stage, with all stages described at length and measured in the paper Bujoczek et al. [1]. There were no signs of recent human intervention in natural forest development processes (dating from the past several decades), but some of the subalpine stands had been managed in the past, as can be inferred from historical records as well as some stumps with saw marks (already in advanced decay stages). Those treatments were probably carried out prior to the establishment of the park. Since then, deadwood has been left in the forest, and phytocoenosis regeneration has occurred naturally.

**Fig. 5:**
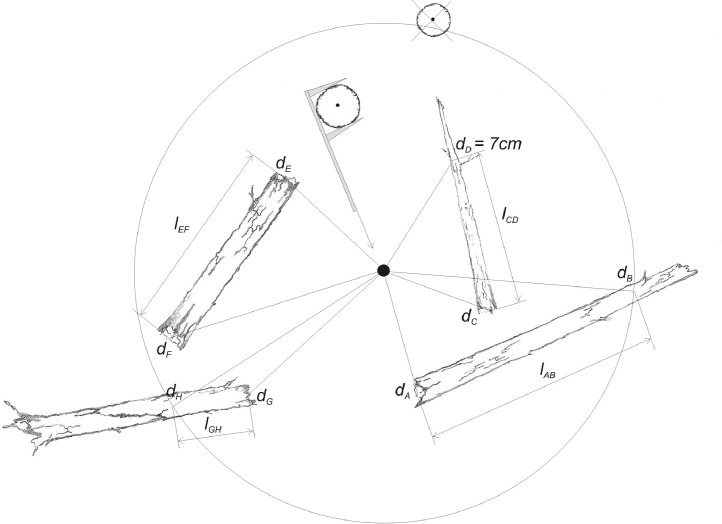
Diagram schematically showing measurements of living trees and deadwood on sample plots.

We established eight sample plots for each of the three development stages to measure characteristics of the tree stands. The centers of the sample plots coincided with the nodes of a 200 *m* × 200 m grid (Fig. 1). After establishing the nodes, we used a Sunnto altimeter to determine the slope of the terrain. Based on the slope, the radii of the sample plots were adopted in such a way that the area of the two concentric circles amounted to 0.05 ha and 0.01 ha, respectively, in a vertical projection on a horizontal plane. We used the 0.05 ha plots for measuring living trees as well as standing or lying dead trees and snags. We included living trees, standing dead trees and snags if their DBH was equal to or larger than 7 cm. Stumps were included if their diameter was equal to or larger than 10 cm at ground level. For a tree to be measured, its center had to be within the radius of the sample plot (Fig. 5). We measured the diameters of snags and stumps above ground and at the top of those tree elements. In the case of high snags, if direct measurement was impossible, the surveyor looked for the broken tip of the tree to determine the top diameter of the snag. Failing that, the top diameter was estimated based on taper. While measuring diameters, the arm of the caliper was directed towards the center of the sample plot. We included all types of downed deadwood (branches, logs, etc.) if their diameter was equal to or greater than 7 cm. In the case of forked fragments, they were divided into segments, with each segment measured separately to ensure accuracy. Similarly as with standing deadwood, we measured downed deadwood only within the boundaries of the sample plots. If a measured fragment of downed deadwood was partially outside the plot, the measurement included only the segment of that fragment up to its intersection with the plot circumference. We measured the diameters of both ends of a downed deadwood fragment, with the surveyor standing and applying the caliper from above. If a deadwood fragment was flattened as a result of decay, we conducted an additional measurement in a direction that was orthogonal with respect to the first one. We characterized each deadwood fragment on a five-point decay scale [1]. If a log exhibited more than one decay stage along its length, it was divided into two or more segments. Deadwood measurement methodology was presented with illustrations in Bujoczek [6].

We evaluated canopy closure visually as percentage ground coverage in a vertical projection on a horizontal plane (on 0.05 ha sample plots). We counted saplings on 0.01 ha sample plots and classified them into one of three size classes defined on the basis of height or DBH as follows: group 1 – 0.5 *m*≤height<1.3 m; group 2 – 0.1<DBH< 3.9 cm; and group 3 – 4 cm<DBH<6.9 cm.

We counted birds across the entire areas of the three forest development stages (30 ha) by means of a combined cartographic method in which the areas were carefully surveyed using transects which were approx. 100 m apart [7]. During the counts, we recorded all sighted or heard birds. Bird location was marked with GPS coordinates to prevent double counting. If possible, we determined the sex and age (adult or juvenile) of the birds. We paid special attention to multiple observations of birds belonging to one species, pairs of birds, and behaviors indicating breeding. Birds passing high in the air were not included. We conducted a total of eight counts in the years 2016–2017: five in the breeding season (March–July) and tree in the nonbreeding season (October–January). The time spent on each study area (forest development stage) during a single count amounted to approx. 5 h.
